# Achieving Source Location Privacy Protection in Monitoring Wireless Sensor Networks through Proxy Node Routing

**DOI:** 10.3390/s19051037

**Published:** 2019-02-28

**Authors:** Lilian C. Mutalemwa, Seokjoo Shin

**Affiliations:** Department of Computer Engineering, Chosun University, Gwangju 61452, Korea; lilian.mutalemwa@gmail.com

**Keywords:** source location privacy, wireless sensor network, patient adversary, cautious adversary, proxy node, random routing

## Abstract

Achieving high source location privacy is critical when Wireless Sensor Networks (WSNs) are used in sensitive applications such as in asset or battlefield monitoring. Due to the sensitivity of information in these applications, it is important to ensure the flow of data between sensor nodes is secure and it does not expose any information about the monitored assets to an adversary. This paper proposes a routing scheme with stronger source location privacy than the privacy of traditional routing schemes. The paper addresses some limitations of four existing schemes by providing highly random routing paths between the source nodes and sink node. The scheme randomly sends packet to the sink node through tactically positioned proxy nodes to guarantee the routes are highly befuddling to the adversary. To achieve high privacy, the proposed scheme uses a randomizing factor to generate a new random route for every successive packet. Simulation results demonstrate that the proposed scheme provides longer safety period and stronger privacy to outperform other schemes. Furthermore the scheme provides stronger privacy against both, patient and cautious adversary models.

## 1. Introduction

The use of Wireless Sensor Networks (WSNs) in monitoring applications can vary from safety-critical monitoring applications such as monitoring of high value assets, the military, healthcare and radiation monitoring, to non-critical applications such as temperature and humidity control [1]. In safety-critical monitoring applications, it is extremely important to ensure that transmission of data between the sensor nodes is secure and information such as location of the asset being monitored is kept private [2,3]. WSNs operate in broadcast mode and packets are routed from the source node to sink node through multi-hop communication. Due to the nature of the sensor node communication in these networks, it is important to keep location information of the source node private because if this information is exposed to an eavesdropping adversary, it will allow the adversary to back trace on the packet routes in the network and locate the source node [2,4]. The process of keeping the location of a source node hidden from an adversary is called source location privacy protection [5]. The source location privacy problem was introduced in [6] using the panda hunter game. In the work of [6], a WSN was deployed to continuously monitor activities and location of the pandas in their habitat. When a node sensed a panda, it became a source node and initiated communication with the sink node to inform about presence of the panda in its monitoring area. The work showed that using a directional antenna, an adversary could monitor the pattern of broadcasts between sensor nodes, identify the location of immediate sender node of the packet and, using this information, it could trace back the packet route to find the ultimate source of the packets and thus the panda [7]. In this work, an alternative approach of defining the source location privacy problem is introduced using battlefield monitoring in military environments. In a battlefield, soldiers may wear sensor nodes which they use to relay packets of information to the sink node. The information in the packets may include details such as their sensor node ID and location or time of packet generation. This information may be very valuable for an eavesdropping adversary. An adversary may use equipment such as spectrum analyzers to trace the packets in the network in a bid to locate the soldiers. The safety of the soldiers maybe very much compromised if the adversary manages to back trace the packet routes to locate the source node which is worn by a soldier. The seminal work in [6] and [8] proposed two essential routing schemes; fake source routing and phantom routing schemes. The schemes have received a lot of attention in the literature and numerous other routing techniques have been proposed to address the source location privacy problem [9].

This paper addresses the source location privacy problem in WSNs by proposing a two-phase random routing scheme that uses strategically positioned proxy nodes to route packets from source node to sink node. In phase 1, packets from a source node are randomly forwarded to a random proxy node located in pre-defined proxy regions. Location of the proxy nodes is designed to give a feeling that source node is sending packets to sink from all possible directions. To make it more difficult for the adversary to predict the routing paths, phase 2 routing is introduced. In phase 2, the proxy node randomly forwards the packet to sink node using random walk routing. Proposed scheme uses a randomization factor and a bias threshold value to guarantee routing paths for each packet are highly random and each packet will use a different route to arrive at the sink node. The highly random routing paths make it a complex task for adversary to predict the route for next packet. The proposed scheme aims to address some limitations of existing source location privacy routing schemes including the shortest path routing [6], phantom single-path routing [8], randomly selected intermediate node routing [10], and all-direction random routing [11] schemes. 

**Figure 1:**
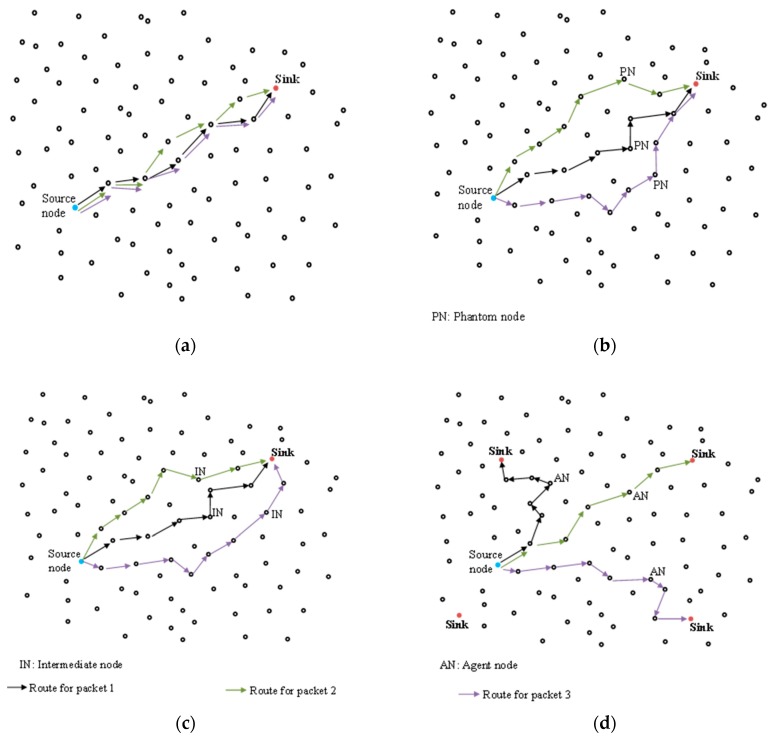
Routing strategies of some existing routing schemes. (**a**) Shortest path routing; (**b**) Phantom single-path routing; (**c**) Randomly selected intermediate node routing; (**d**) All-direction random routing.

The routing strategies and limitations of some of the existing routing schemes are demonstrated in Figure 1. Figure 1a shows the routing strategy for the shortest path routing. In this scheme, packet forwarding algorithm employs a single shortest path between source node and sink. The routes for packets 1, 2 and 3 are very closely related. The use of shortest paths provides poor privacy since it is easy for adversary to back trace the short routes and locate the source node within a short time. The closely related routes make it easy for adversary to guess the routes for successive packets. Figure 1b shows the routing strategy for the phantom single-path routing. In this scheme, the source node sends packet to a random phantom node through random walk routing then uses a single fixed path to deliver packets from the phantom node to sink node. The single fixed path is implemented using the shortest path routing strategy. The use of shortest path routing between the phantom node and sink node allows the adversary to easily back trace to the phantom node and eventually to the source node. In Figure 1b, the shortest routing paths between the phantom nodes and sink become closely related and more predictable. Furthermore, the scheme always obeys a constant rule for routing packets without considering if the source node is near sink node or not. The routes become more predictable to the adversary if a source node is near the sink node [11]. Figure 1c shows the routing strategy for the randomly selected intermediate node routing scheme. This scheme pre-defines a region around the source node and randomly selects an intermediate node located outside the pre-defined region. The source node sends packet to the selected intermediate node through random walk routing then the intermediate node forwards the packet to sink node through a fixed route. The use of fixed routes between the intermediate nodes and sink node reduces the privacy level of the scheme since it is easy for adversary to back trace a fixed route. It takes a short time for adversary to back trace the fixed route to locate the intermediate node and eventually the source node. Moreover, the scheme employs a constant rule for routing packets without considering the location of the source node. If a source node is near the sink, there is a high probability of the selected intermediate node to be away from the source node but very near the sink node to create a short routing path and poor privacy. Figure 1d shows the routing strategy for the all-direction random routing scheme. This scheme is one of the recently proposed schemes. In the quest to generate random routes, it employs multiple sink nodes and a packet is randomly forwarded to one of the sink nodes through agent nodes. The use of multiple sink nodes requires the sink nodes to further communicate directly so as to synchronize the packet information. The additional direct communication between sink nodes results in increased packet delivery costs such as packet delivery latency and energy consumption while reducing the packet delivery ratio. Furthermore, the scheme has a high probability of successive packets to be routed through the same agent node hence reduced privacy. Table 1 summarizes the key features, limitations and routing strategies of the existing routing schemes. It also highlights the strategies for improvement in the proposed scheme.

Specifically, the main contributions of this paper can be summarized as follows: (1) to propose a new routing scheme that uses strategic proxy nodes to provide strong source location privacy for source nodes in all areas of the network; (2) to conduct a series of experiments to evaluate performance of the proposed routing scheme against patient and cautious adversaries; (3) to demonstrate that the proposed scheme provides stronger source location privacy than some of the existing schemes including the shortest path routing, phantom single-path routing, randomly selected intermediate node routing, and all-direction random routing schemes. 

The remainder of this paper is organized as follows: Section 2 presents a review of the literature on routing schemes for source location privacy. Section 3 gives details of the network and adversary models. The proposed routing scheme is described in details in Section 4. Privacy analysis of the proposed scheme is provided in Section 5 and performance analysis with the simulation results are presented in Section 6. In Section 7, the paper is concluded.

## 2. Related Work

The formalization of source location privacy problem in [6] and [8] explored the widely adopted shortest path routing, fake source routing, and phantom source routing schemes. In the shortest path routing, a source node creates a single route to the sink node using a gradient-based approach. During packet forwarding, a node which has the shortest hop-distance to the sink is assigned the maximum gradient and packets are always forwarded to the next-hop node which has the maximum gradient. The scheme has tradeoffs between privacy, energy consumption and packet delivery performance. The shortest routes provide a short safety period and very poor privacy while consuming very low energy and deliver packets with the lowest delay and highest delivery ratio. The baseline fake source routing scheme uses a set of fake source nodes to act as real sources. The fake sources generate packets to model the network traffic in a way that confuses an adversary by leading it away from the real source. The fake packets are of the same length as the real packets, and they are encrypted so as to make it difficult for adversary to tell the difference between a fake packet and a real packet. Several versions of fake source routing exist including the short-lived fake source routing, persistent fake source routing, dynamic fake source routing and a distributed solution that combines fake source routing and phantom routing [12]. The most significant limitation of many fake source routing schemes is their very high energy consumption due to the high volume of packets required to broadcast in order to provide effective source location privacy. Moreover, the high volume of packets in the network has a negative effect on the packet delivery ratio of the routing schemes due to increased number of packet collisions. More packet flow in the network increases the probability for packet collisions. The work of [2] and [13] are among schemes with strategies to improve the limitations of fake source routing. In [2], a hybrid online algorithm that uses directed random walks for the fake sources allocation was introduced to reduce the energy consumption of the scheme. The algorithm also reduces the number of packet collisions and improves the delivery ratio of the scheme by lowering the number of fake packets in the network. In [13], the relationship between real and fake source broadcast rates and the number of packet collisions was highlighted showing that there exist practical rates at which source nodes should broadcast for the scheme to be energy efficient. Phantom routing scheme was introduced to improve the limitations of fake source routing scheme. Phantom routing is a two-phase routing scheme where packets are first forwarded to a random phantom source through random walk, and then, a succeeding flooding or single-path routing is used to forward the packets to sink node. Phantom single-path routing [8] is more efficient than phantom flooding [6] in terms of safety period and energy consumption. Limitations of phantom source routing include low privacy especially for source nodes located near the sink node. Several versions of phantom routing exist including the directed walk phantom single-path routing [6], phantom routing with locational angle [14], and the greedy random walk routing [15]. 

The randomly selected intermediate node routing scheme was introduced in [10]. The scheme randomly selects an intermediate node using a pre-defined constrained region around the source node. The scheme operates in two phases where packets are first forwarded to an intermediate node located at a determined minimum distance from the source node, then, the intermediate node forwards the received packet to the sink node. The scheme provides a somewhat better privacy than phantom routing but at slightly higher packet delivery cost such as energy consumption and packet delivery latency. Similar to phantom routing, this scheme provides poor privacy for source nodes near sink. Some routing paths get shorter as the source node gets closer to sink node. A three phase intermediate node routing scheme was proposed in [16]. The scheme uses a mixing ring region which is placed between an intermediate node and the sink node, and a packet is routed through a node in the region. A packet traveling in the mixing ring region is mixed with other packets. The main limitation of this scheme is the unbalanced energy consumption in the network, because ring nodes are more likely to deplete their batteries faster than the other nodes. Two of the recently proposed intermediate node-based random routing schemes are; all-direction random routing [11], and strategic location-based random routing [5] schemes. The all-direction random routing scheme uses a three phase routing strategy and employs multiple sink nodes. The source node first selects a proper sink node and agent node to route the packets, then, the source node forwards the packets to the agent node. In the agent node selection process, the source node first selects a location *L* near to the selected sink node and a node nearest to location *L* is randomly selected as the agent node. In the third phase, the selected agent node forwards the packet to the sink node. The strategic location-based random routing scheme uses a two phase routing strategy. Depending on the location of the source node, a random diversion node or mediate node is selected for routing the packets to the sink node. Both schemes provide strong source location privacy protection. Their privacy performance is much higher than the traditional routing schemes. The limitations of the schemes include higher energy consumption and delivery latency with a reduced packet delivery ratio as compared to the traditional routing schemes.

A routing scheme can also be designed to route packets in the network in a tree-based or angle-based routing strategy. The tree-based diversionary routing scheme [14] is one example of the tree-based routing schemes. It operates in two phases. In the first phase, it creates a backbone routing path directly to the network border based on phantom routes. In the second phase, it establishes many redundant diversionary routes in regions far away from the sink node to divert the adversary from the real packet route. The scheme also employs fake source nodes at the end of the diversionary routes to confuse the adversary. The scheme provides much higher source location privacy protection as compared to many existing routing schemes. The main limitation of the tree-based diversionary routing scheme is the very high energy consumption due to the extremely long routing paths which divert to the network border and the use of fake packet sources. The angle-based dynamic routing scheme [17] uses location information of the nodes and calculates two inclination angles formed between the nodes. The two angles are; the inclination angle between a forwarding node and a receiving node, and the inclination angle between a forwarding node and the sink node. A candidate set of neighboring nodes is generated based on the angles. The candidate set changes at every packet forwarding instance to form dynamic paths towards the sink node. The recently proposed angle-based routing schemes include the constrained random routing [18] and the 2-phantom angle-based routing [19]. Constrained random routing is based on the transmitting offset angles and the constrained probability. To prevent adversary from tracing back to the source node location, first, each forwarding node determines a specific selection domain for next-hop node according to the dangerous distance and the wireless communication range. Subsequently, it analyzes the offset angles of the candidate nodes based on the direction of the nodes to the sink node. Lastly, the forwarding node calculates the selected weights of the candidate nodes according to their offset angles. The selected weights are used to decide which node to become the next-hop node until packet reaches at the sink node. The lengths of routing paths are controlled by giving greater priority to the nodes with smaller offset angles. The 2-phantom angle-based routing scheme considers a triplet for selecting the phantom nodes. A triplet is a group of three nodes formed on the basis of three parameters; their distance from the sink node, their location information, and the inclination angle between them. Phantom selection is performed for every packet forwarding instance to create dynamic routing paths for the packets.

## 3. Models

In this section, the relevant features of the proposed network and adversary models are introduced and assumptions are highlighted.

### 3.1. Network Model

The assumed network model is a two-dimensional network composed of a set of sensor nodes and links. A wireless sensor node is a computing device equipped with a wireless interface, limited set of computational capabilities and a unique identifier (ID). Three types of sensor nodes and sensor node functionalities exist in the network; sink node, source node and normal (relay) nodes. Sink node is responsible for collecting data from the other nodes and it acts as a link between the WSN and the external world. The sink node is more powerful than the other nodes. It has more memory capacity and more computational power. The source node is responsible for sensing the asset and forwarding the sensed data to sink node through multi-hop communication. Normal nodes are used to relay packets from the source node to sink node. Communication from a node is typically modelled with a circular communication range centered at the node. All nodes are homogeneous and have the same communication range. Nodes in direct communication range with each other through 1-hop communication are considered neighboring nodes and are able to exchange data. Network is event-triggered, i.e., when a source node senses an asset, it starts sending packets periodically to the sink node. When a node detects an asset in its monitoring area, it remains active until the asset moves out of its monitoring area. When the asset moves to a new location, it triggers another sensor node to become the new source node. When no asset is detected, the nodes may follow a sleeping schedule. Transmitted packets are encrypted and contain source node ID which only the sink can infer as an asset location. Network uses the network initialization process for localization of sensor nodes where each node can locate itself, can easily learn its neighboring node locations and IDs and learn the location of the sink node. The network employs the k-nearest neighbor tracking approach to track the assets.

### 3.2. Adversary Model

Strength of an adversary can be factored along two dimensions: presence and actions [1,3]. Presence is concerned with network coverage of the adversary while action is concerned with type of attacks it can launch. In this work, the adversary is assumed to be well equipped and has sufficient resources such as adequate computation capabilities, memory and unlimited power. It is equipped with antenna and spectrum analyzers, so it can observe the wireless communication within a certain detection range. The adversary is mobile, initially residing in the vicinity of the sink node listening for arriving packets. Adversary starting at the sink node guarantees it hears packets since sink node is the destination for all packets. When a packet is received at the sink, the adversary will overhear and start back tracing the packet route by moving 1 hop towards the source node until it reaches the source node. The adversary has knowledge of the routing strategy in the network according to Kerckhoff’s principle and has the same transmission range as that of sensor nodes.

On detecting a packet, adversary can measure the angle of arrival of the signal and the received signal strength to identify the immediate sender node and perform back tracing attack by moving to the immediate sender node location without any delay. Once at the immediate sender node, the adversary keeps on listening on the communications between the node and its neighboring nodes and continues to back trace to the source node. The adversary never misses a packet when it is within the transmission range of the receiving node. Based on the adversary capabilities, several adversary models can exist as explained in [9]. This work considers two main categories of the adversary models; passive adversary, and active adversary. A passive adversary performs only passive attacks such as simply eavesdropping on sensor communication and does not interfere with the normal operations of the network. Active adversaries are highly motivated and can interfere with the normal operation of nodes by injecting, modifying, or blocking packets from a portion of the network or by reprogramming the sensor software [1,9]. A passive adversary is the most commonly assumed adversary type during designing of routing schemes for source location privacy [9,20]. Active adversaries are less common because they have more chances of being caught by the network operator if they interfere with the normal operation of the WSN. Four main types of passive adversary models can exist: (1) patient, local adversary; (2) cautious, local adversary; (3) global adversary; and (4) direction-oriented adversary [20].

The patient and cautious adversary models are the most commonly assumed adversary models. The models were assumed in the seminal works of [6] and [8] and numerous other works have adopted the models. The direction-oriented adversary model was introduced in [21] and it has not been widely adopted. This work assumes the two widely adopted adversary models; the patient, local adversary and cautious, local adversary models.


**Algorithm 1. Patient Adversary Algorithm**
1:Adversary_location = Sink_location2:When adversary overhears a packet
Adversary_location = Immediate_sender_node_location3:**while** (Adversary_location! = Source_node_location)**do**
4:   Adversary_location = Immediate_sender_node_location5:
**end while**

// Source_node _location found

A patient adversary patiently waits at a node until it hears a packet and moves to the immediate sender node of the packet. It repeats the process until it finds the immediate sender node is the source node and locates the asset. In the cautious adversary model, the adversary will use a timer to limit its waiting time at a node. If the timer expires, the adversary will roll back to its previous node and resume packet listen at that node. Furthermore, a cautious adversary will store information of all visited immediate sender nodes to avoid revisiting nodes which have already been visited to escape from getting trapped in a loop. The back tracing algorithms for the patient and cautious adversaries are presented in Algorithm 1; Algorithm 2, respectively.


**Algorithm 2. Cautious Adversary Algorithm**
1:Adversary_location = Sink_location2:Adversary stores information of all the Visited_immediate_sender_nodes in its memory3:When adversary overhears a packet
Adversary_location = Immediate_sender_node_location4:At the Adversary_location, wait for a fixed amount of time as set on the timer5:**while** (Adversary_location ! = Source_node_location) **do**
6:    **if** (packet arrives at Adversary_location from Immediate_sender_node before timer expires) **then**
7:      **if** (Immediate_sender_node ! = Visited_immediate_sender_nodes) **then**8:       Adversary_location = Immediate_sender_node9:       Update Visited_immediate_sender_nodes with Adversary_location10:      **else**11:       discard the packet 12:      **end if**
    **else**13:      move to the last Visited_immediate_sender node.14:    **end if**15:
**end while**

// Source_node _location found

## 4. Proposed Proxy Node Routing Scheme

**Figure 2:**
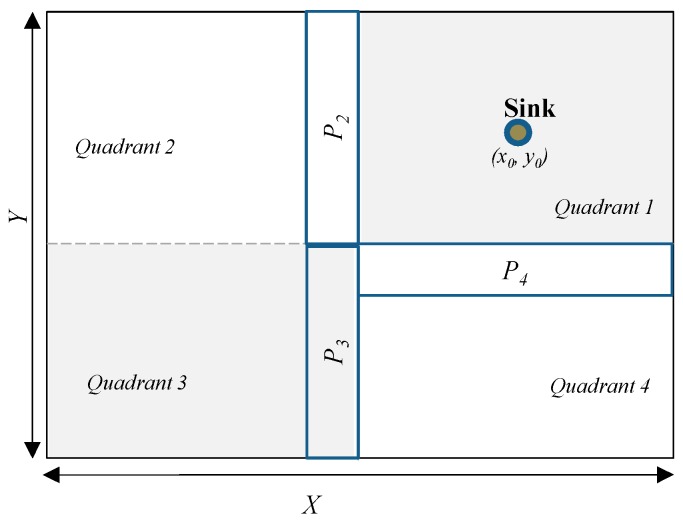
Division of the WSN domain.

**Figure 3:**
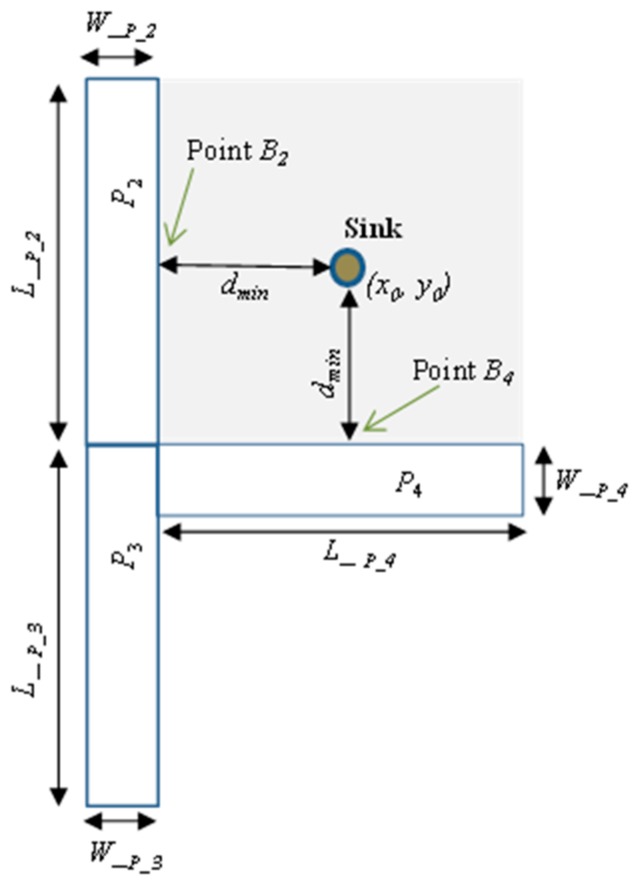
Configuration of the proxy regions.

The proposed scheme assumes the following; the sensor domain is divided into four quadrants. *Quadrants 1*, *2*, *3* and *4* and their positions are at the right-up, left-up, left-down and right-down, as shown in Figure 2. *X* and *Y* are the length and width of the WSN domain. Sink node is located at (*x*_0_, *y*_0_) in *Quadrant 1* while proxy nodes are strategically located in proxy regions *P*_2_, *P*_3_ and *P*_4_ in *Quadrants 2*, *3* and *4*, respectively. Locating the sink in *Quadrant 1* is good for applications such as in battlefield monitoring or in ocean communications where the sink node (base station) is located towards the network edge. Proxy regions are located at the boundaries of *Quadrants 2*, *3*, and *4* nearest to *Quadrant 1*. The configuration of the proxy regions is designed in such a way that the minimum distance between sink node and point *B*_2_ at the border of proxy region *P*_2_ and point *B*_4_ at the border of proxy region *P*_4_ is equal to *d_min_* as shown in Figure 3. The distance *d_min_* can be calculated using the Euclidian distance equation. For example, if location of point *B*_2_ is (*x*_*b*2_, *y*_*b*2_), *d_min_* is then determined as: $d_{min}=\sqrt{{\left(x_{b2}-x_{0}\right)}^{2}+{\left(y_{b2}-y_{0}\right)}^{2}}$. Distance from the sink node to a proxy node within proxy regions *P*_2_, *P*_3_ or *P*_4_ is *d_proxy_*. The value of *d_proxy_* will always satisfy the equation: $d_{proxy}\geq d_{min}$.

This location of the proxy regions is considered to ensure three main design objectives: (1) to guarantee that proxy nodes are far away from the sink node at a minimum distance *d_min_* to make it difficult for adversary to back trace from sink node to a proxy node; (2) to increase the length and randomness of the routing paths for source nodes in *Quadrant 1*; and (3) to control the packet delivery latency and energy consumption specifically for source nodes in *Quadrant 1*. If proxy nodes are too far away from the boundary of *Quadrant 1*, the delivery latency and energy consumption will be increased. 

A source node can be in any of the four quadrants and two sets of proxy nodes are available for each source node. A source node selects a proxy region of a quadrant other than its own. For instance, source nodes in *Quadrant 1* will randomly select proxy nodes from proxy regions *P*_2_ or *P*_4_. Source nodes in *Quadrant 2* will randomly select proxy nodes from proxy regions *P*_4_ or *P*_3_. Source nodes in *Quadrant 3* will randomly select proxy nodes from proxy regions *P*_4_ or *P*_2_. Source nodes in *Quadrant 4* will randomly select proxy nodes from proxy regions *P*_3_ or *P*_2_. The proxy node selection process according to source node location is summarized in Table 2. The table is explained more in the next paragraph. Selecting proxy nodes according to the source node location offers a dynamic route creation process as opposed to the shortest path routing, phantom single-path routing and randomly selected intermediate node routing schemes which apply constant rule throughout the network. This dynamic route creation process highly improves the source location privacy for source nodes located in near-sink regions as explained in the next section.

In this paper, it is assumed all proxy regions *P*_2_, *P*_3_ and *P*_4_ have equal coverage areas. Therefore, the lengths (*L__P_*) of all proxy regions are equal and the widths (*W__P_*) of all proxy regions are equal as shown in Figure 3. That is, $L{\_}_{P\_2}=L{\_}_{P\_3}=L{\_}_{P\_4}$ and $W{\_}_{P\_2}=W{\_}_{P\_3}=W{\_}_{P\_4}$. The size of proxy regions and distance *d_min_* are determined by the network operator during the network initialization phase. The size of proxy regions is carefully designed to ensure availability of an effective number of proxy nodes. To guarantee the routing paths are highly random for each packet, a source node generates a randomization factor, *R_F_*, which is distributed between 0 and 1 and compares it with a pre-defined threshold, *T*. A proxy node is randomly selected from one proxy region or the other depending on whether *R_F_* is less than threshold *T* or not as shown in Table 2. For example, for source node in *Quadrant 1*, if *R_F_* = 0.3 and threshold *T* = 0.5, then *R_F_* is less than *T* and therefore the source node will randomly select one proxy node from region *P*_2_. If the *R_F_* for a successive packet is 0.7 which is greater than *T* = 0.5, one proxy node is randomly selected from region *P*_4_. All proxy nodes in regions *P*_2_, *P*_3_ and *P*_4_ have equal probability of being selected as a random proxy node. Operation of the proposed scheme begins with the network initialization process and thereafter packet routing.

### 4.1. Network Initialization

It is assumed that the network initialization process will begin by the network operator determining the configuration of the WSN domain according to Figure 2; Figure 3 including the value of *d_min_*. Then, each node takes part in the process of learning its own location, location of the neighboring nodes and of the sink node. The process starts when each sensor node is loaded with a unique ID. Sink node obtains its location information using Global Positioning System (GPS). Sink node then broadcasts a beacon packet to all sensor nodes in the network and sets its hop counter to zero. Each node receives the beacon packet, stores the hop counter value with sender node ID, adds the hop counter by one and rebroadcasts the beacon packet to its neighboring nodes. The hop counter number represents the hop-distance of the sensor node from the sink node. This process gives each node a good knowledge about its neighboring nodes and its location and distance with respect to the sink node. When a sensor node receives multiple packets, it only stores the minimum hop count in its buffer and deletes other hop counter information. After that, each sensor node calculates and records a set of its neighboring nodes. Each node informs its hop-distance to the sink node. At the end of the network initialization process, the sensor nodes located in the regions of *Quadrants 1*, *2*, *3* and *4*, and proxy nodes in regions *P*_2_, *P*_3_ and *P*_4_ are identified.

### 4.2. Packet Routing Strategy

Packet routing of the proposed scheme begins when a source node detects the asset. Upon detection of the asset, the source node generates encrypted packets to send to the sink node through multi-hop routing. The routing strategy is two-phase routing. In phase 1, source node randomly sends packet to a randomly selected proxy node. In phase 2, the selected proxy node forwards the packet to the sink node through random walk routing.

#### 4.2.1. Phase 1

Upon detection of the asset, the source node calculates *R_F_* and compares it to *T*. One proxy node is randomly selected from proxy regions according to source node location and the value of *R_F_* as illustrated in Table 2. The source node then determines a group of neighboring nodes with shorter hop-distance to the selected proxy node. One neighboring node from the group is randomly selected as the next-hop node. The source node randomly forwards the packet to the next-hop node and eventually to the selected proxy node. The random choice of one proxy node out of two proxy regions for each packet makes the routing paths more random, increases the complexity for the adversary to predict the route for next packet, and improves the source location privacy. Phase 1 routing ends when packet reaches the proxy node.

#### 4.2.2. Phase 2

Upon reception of the packet from the source node, the proxy node determines a group of neighboring nodes with shorter hop-distance to the sink node. One neighboring node from the group is randomly selected as the next-hop node. The proxy node randomly forwards the packet to the next-hop node and eventually to the sink node through random walk routing.

**Figure 4:**
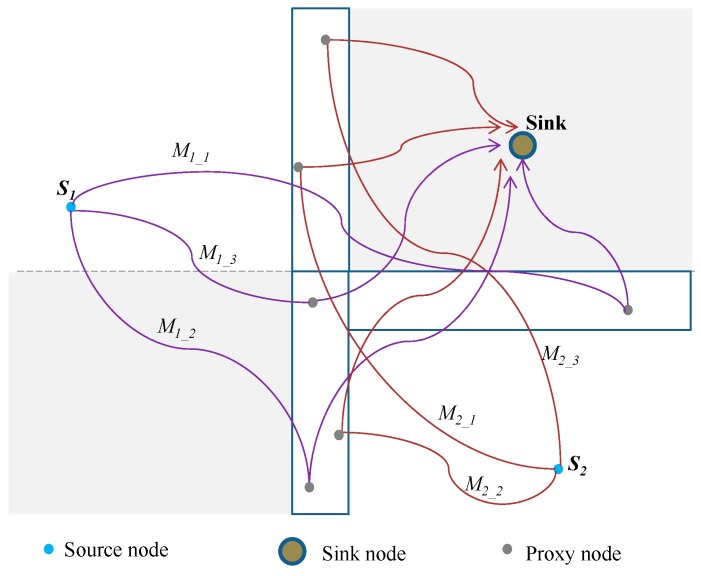
Example packet routing using the proposed proxy node routing scheme.

Figure 4 shows an example packet routing using the proposed routing scheme. In this example two source nodes exist, source nodes *S*_1_ and *S*_2_. *S*_1_ is located in *Quadrant 2* while *S*_2_ in *Quadrant 4*. Each source node sends 3 packets consecutively. The figure demonstrates that the proposed scheme has the capacity to send the packets through routing paths which are created in different regions of the network so as to confuse the adversary. For *S*_1_, packets *M_1_1_*, *M_1_2_* and *M_1_3_* are sent through different proxy regions *P*_3_ or *P*_4_ while for *S*_2_, *M_2_1_*, *M_2_2_* and *M_2_3_* are sent through proxy regions *P*_2_ or *P*_3_. The proposed scheme guarantees high probability that successive packets are routed through proxy nodes which are spaced out throughout the proxy regions. If proxy nodes for successive packets are far from each other as shown in the figure, it becomes easier for a cautious adversary to get trapped in a loop and make less progress towards the source node. For a patient adversary, it will take a longer time to receive a packet at the same node again, reducing its progress towards the source node. For example, for source node *S*_1_, *M_1_1_* is routed through proxy node located on the right side of region *P*_4_ while *M_1_2_* on the bottom part of region *P*_3_ and *M_1_3_* on the top part of region *P*_3_. It is obvious that even if the adversary locates the proxy node for *M_1_1_*, it gets no help in predicting the location for *M_1_2_* or *M_1_3_* proxy nodes. The routes are very diverse and highly perplexing.

## 5. Privacy Analysis

In the proposed scheme, a source node routes packet to the sink node through proxy nodes selected from the sensor domain in proxy regions *P*_2_, *P*_3_ or *P*_4_. In shortest path routing and phantom single-path routing, a source node in *Quadrant 1* will use a short route to deliver packets to sink. The short routes may allow the adversary to successfully back trace to the source node in a short time. Also, short routes increase the chances of packets from same source node to use closely related routing paths where adversary is likely to receive successive packets and easily back trace to the source node. In the proposed scheme, a source node in *Quadrant 1* will first send the packet to a random proxy node in regions *P*_2_ or *P*_4_ and the proxy node will randomly forward the packet to sink node. The random routes through proxy regions elongate and diversify the routes to give stronger privacy. Highly random routing paths reduce the chances of adversary to capture successive packets, increase the complexity for adversary to guess the route for next packet and improve the source location privacy.

The proposed scheme provides highly random routes as compared to that of randomly selected intermediate node routing and all-direction random routing schemes. In the randomly selected intermediate node routing, the fixed routes between the intermediate nodes and sink nodes creates predictable routes that can lead the adversary to the source node and jeopardize the privacy level of the scheme. The use of randomization factor *R_F_* for each packet in the proposed scheme enables the routes between proxy nodes and the sink node or proxy nodes and source nodes to highly obscure the adversary. Furthermore, the use of *R_F_* for each packet enables the proposed scheme to dynamically select proxy nodes in different regions of the WSNs to allow successive packets to use more diverse routing paths as compared to the routing paths of the all-direction random routing scheme. The proposed scheme has a much lower probability of successive packets to select same proxy node while the all-direction random routing has high probability of successive packets to select same agent node. If the probability of successive packets to select same agent node is high, the routes become more predictable to the adversary and privacy level is reduced. Overall, the proposed scheme provides stronger source location privacy for source nodes in all four quadrants by employing a diverse routing strategy according to the source node location. It does not employ a constant rule throughout the network as it is done in shortest path routing, phantom single-path, and randomly selected intermediate node routing schemes. 

The proposed scheme performs well against the two adversary types: patient and cautious adversaries. For successful back tracing attack to the source node, an adversary needs to intercept many packets. If packets in the proposed scheme use highly random routing paths through the proxy nodes, it will take longer for the adversary to capture enough packets to successfully locate the source node. The adversary might find itself using a longer time and possibly the asset will move to a new location before the adversary locates the source node. Considering a patient adversary who waits patiently at a node, adversary will make insignificant progress towards the source node in the proposed routing scheme as compared to the shortest path routing, phantom single-path routing, randomly selected intermediate node routing or all-direction random routing schemes. The highly random routing paths for each packet will cause a patient adversary to wait for a much longer time at a node before it receives a packet again. In the case of a cautious adversary, if packets don’t arrive at a node within the set waiting timer, adversary might find itself rolling back to previous immediate sender nodes and making insignificant progress towards the source node.

## 6. Performance Analysis

### 6.1. Simulation Environment

Performance analysis to evaluate the performance of the proposed scheme was done using MATLAB network simulation. In the simulation, the target area was a square grid network layout of size 2000 × 2000 m^2^. 2500 nodes were randomly distributed in the target area. Sink node was the destination for all the packet transmissions. The sink node was assumed to be located at the center of *Quadrant 1* as explained in Section 4. Adversary was initially deployed around the sink node and it performed hop-by-hop back tracing attack to find the location of the source nodes. To ensure high privacy with controlled packet delivery delay and energy consumption as explained in Section 4, proxy regions *P*_2_, *P*_3_ and *P*_4_ were located at quadrant boundaries nearest to *Quadrant 1* following the distribution shown in Figure 2 and Figure 3. Proxy regions had equal coverage areas. To guarantee availability of an effective number of proxy nodes in the network, length and width of the proxy regions were set as follows: *L__P_2_* = 0.5*Y*, *L__P_3_* = 0.5*Y* and *L__P_4_* = 0.5*X*. *W__P_2_* = 0.2*X*, *W__P_3_* = 0.2*X*, and *W__P_4_* = 0.2*Y*.

**Figure 5:**
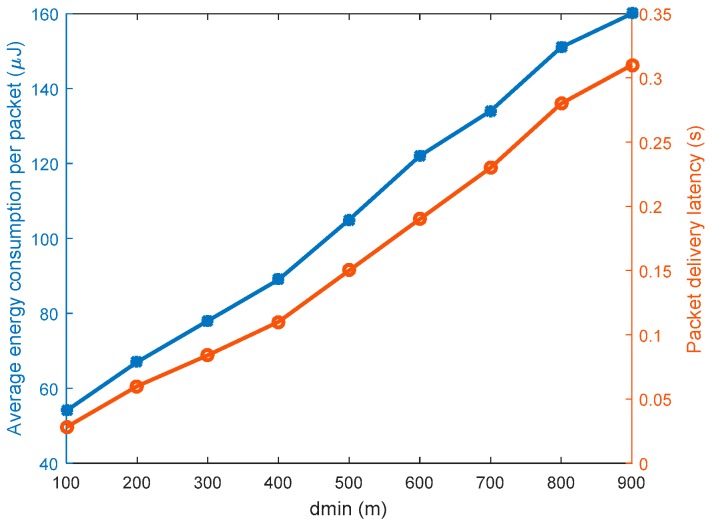
Energy consumption and packet delivery latency performances for various *d_min_* values.

A thorough analysis to determine the value of *d_min_* was done using two performance metrics; energy consumption and packet delivery latency. Figure 5 show the energy consumption and packet delivery latency performances for the source nodes in *Quadrant 1* as *d_min_* is varied. The analysis used source nodes in *Quadrant 1* since the value of *d_min_* has the highest impact on the energy consumption and packet delivery latency for source nodes in *Quadrant 1* as compared to the other quadrants. While routing packets for source nodes in *Quadrant 1*, the packets are first diverted to the proxy nodes located at minimum distance *d_min_* from the sink node before they are forwarded to the sink node. If *d_min_* is too long, the routes become too long and significantly increase the energy consumption per packet delivery and the packet delivery latency. The figure shows that energy consumption per packet delivery and packet delivery latency performances have a directly proportional relationship with the value of *d_min_*. Energy consumption per packet delivery increases with increase in *d_min_*. Similarly, packet delivery latency increases with increase in *d_min_*. A network operator may determine the optimum value of *d_min_* following the performance in Figure 5. In this work, the value of *d_min_* = 500 m is considered because at this point, the energy consumption per packet delivery and packet delivery latency are at roughly the average values. Additionally, this value of *d_min_* = 500 m has the benefits of dividing the quadrants into 4 equal coverage areas considering the network size as summarized in Table 3. The pre-defined threshold *T* was set at 0.5. The WSN employed the k-nearest neighbors tracking approach to monitor the target area. Simulation was run for 500 iterations and average values were considered. 

The simulation environment explained above was used for performance analysis of the proposed scheme, shortest path routing, phantom single-path routing, randomly selected intermediate node routing, and all-direction random routing schemes. The analysis also included our previously proposed scheme, the strategic location-based random routing scheme. Privacy performance analysis of the schemes against both, patient and cautious adversary was done. The random walk of the phantom single-path routing scheme was set to 20% of the hops between the source node and the sink node. The number of sink nodes in the all-direction random routing scheme was set to 4. One sink was located at the center of each quadrant. For cautious adversary, waiting timer was set to 4 source packets. The network simulation parameters are summarized in Table 3. The sensor node sensing range was set to 30 m to ensure multi-hop communications between source nodes and sink node. Adversary detection range was set to 30 m similar to sensor node sensing range to ensure adversary performs hop-by-hop back tracing attack. 

**Figure 6:**
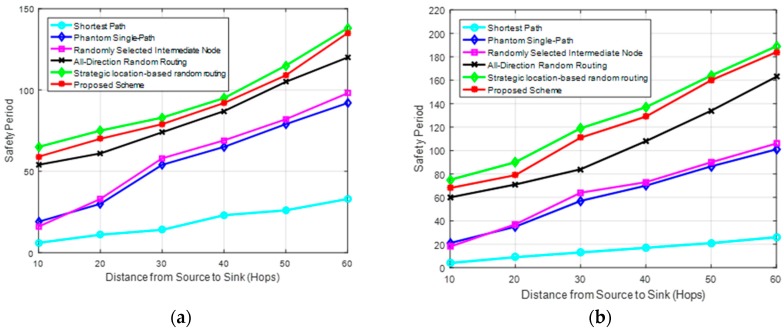
Privacy performance of the routing schemes. (**a**) Safety period of routing schemes against a patient adversary; and (**b**) Safety period of routing schemes against a cautious adversary.

**Figure 7:**
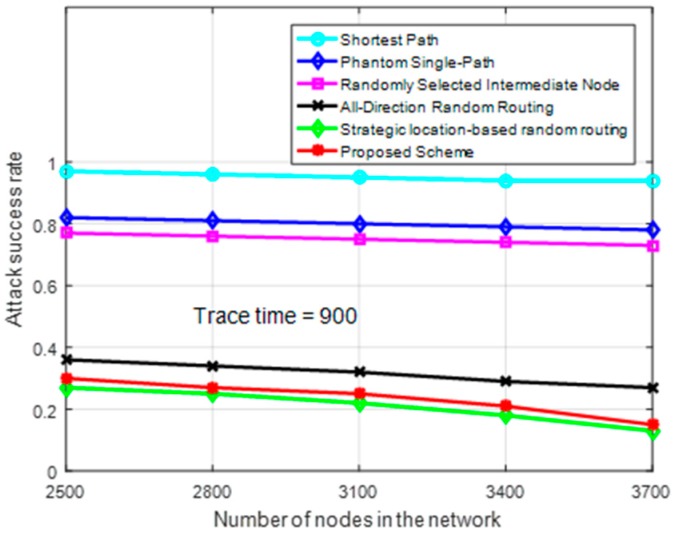
Attack success rate for different number of nodes in the network.

**Figure 8:**
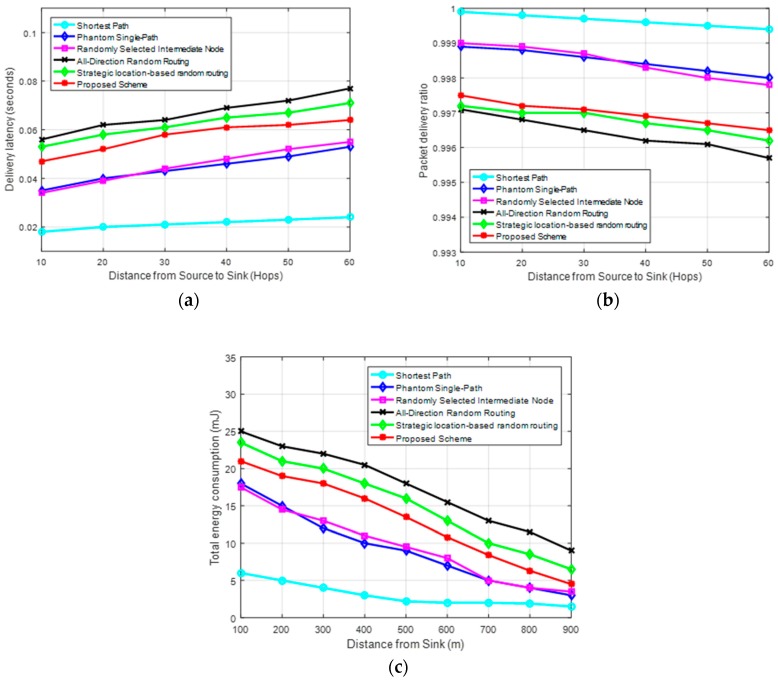
Performance of the routing schemes. (**a**) Packet delivery latency; (**b**) Packet delivery ratio; and (**c**) Energy consumption.

The energy consumption model was adopted from [5,21]. To transmit an *l*-bit packet to a transmission distance *d*, transmission energy, *E_t_*, and receive energy, *E_r_*, follow Equations (1) and (2), respectively. In this model, the energy consumption for packet transmission is proportional to *d*^2^. *E_elec_* denotes transmitting circuit loss. The model uses both, the free space (*d*^2^ power loss) and the multi-path fading (*d*^4^ power loss) channel models. If the transmission distance is less than the threshold *d*_0_, the power amplifier loss is based on free-space model; otherwise, the multi-path attenuation model is used. *E_fs_* and *E_amp_* are the energy required by power amplification in the two power loss models. Simulation results in Figure 6, Figure 7 and Figure 8 demonstrate the performances of the schemes. Performance metrics including the safety period, attack success rate, packet delivery latency, packet delivery ratio, and energy consumption were used for analysis:

(1) $$E_{t}=\{\begin{array}{l} lE_{elec}+lE_{fs}d^{2},\;if\;d<d_{0}\\ lE_{elec}+lE_{amp}d^{4},\;otherwise \end{array}$$

(2) $$E_{r}=lE_{elec}$$

### 6.2. Simulation Results and Discussions

Figure 6a,b show the safety period of the schemes against the patient and cautious adversaries, respectively. Safety period is a performance metric used to measure privacy level of a routing scheme. Safety period can be defined in two ways [3]. It can be defined as the time required for an adversary to back trace and capture the asset. Or, it is the maximum time an asset will be at a given location before it moves to a new location. This work assumes the first definition. Longer safety period provides higher privacy level. The proposed scheme has longer safety period as compared to the shortest path routing, phantom single-path routing, randomly selected intermediate node routing, and all-direction random routing schemes. The scheme provides longer safety period in all regions of the network, including for source nodes in near-sink or far from sink regions. The longer safety period is due to two reasons: (1) the use of randomization factor *R_F_* which guarantees highly random routing paths for successive packets; and (2) the use of elongated routing paths which are created through the randomly selected proxy nodes. The routing paths of the proposed scheme also improve path diversity by allowing individual packets to follow different routes to the sink node. This has a positive effect on the privacy level by making it more difficult for the adversary to guess the route for the successive packet. The all-direction random routing scheme has lower safety period than the proposed scheme because the routes are less random, and there is a higher probability of the successive packets to select the same agent node nearest to the selected virtual location *L*. If the successive packets select the same agent node, the routing paths become more predictable to the adversary. The phantom single-path routing and randomly selected intermediate node routing have almost comparable performances. Both of these schemes deliver packets to the sink through a fixed route from the phantom or intermediate node. Using a fixed single path between the sink node and phantom or intermediate node lowers the privacy level by making it easy for adversary to back trace to these nodes and eventually to the source node. Furthermore, these schemes have a higher probability of the selected intermediate or phantom nodes to be very near the sink node if a source node is located near the sink node. Selecting a phantom or intermediate node which is located near the sink node will lead to poor privacy since it will take a short time for adversary to back trace to the nodes. The shortest path routing has the lowest safety period because it uses the shortest paths to deliver packets to the sink node. Adversary can easily back trace the shortest paths in a short time. Safety period for all schemes increases as the distance from source node to sink node increase since the routing paths become longer as distance from the sink increases. The proposed scheme and the strategic location-based random routing scheme achieve a somewhat comparable privacy level while the proposed scheme is more cost-effective as depicted in Figure 6, Figure 7 and Figure 8.

Figure 6a,b also demonstrate that the proposed routing scheme has a much longer safety period against a cautious adversary as compared to when it is used against a patient adversary. The use of randomization factor *R_F_* and strategically positioned proxy nodes makes the routing paths more unpredictable and packets seem to arrive at the sink node from all possible directions. It becomes easier for the cautious adversary to be trapped in a loop and make less significant progress towards the source nodes. As an example, in Figure 4, if a cautious adversary tries to back trace the successive packets *M_2_1_*, *M_2_2_* and *M_2_3_* from the source node *S*_2_, it is highly possible that the waiting timer will expire and adversary will find itself rolling back to previous immediate sender nodes and make small progress towards the source node *S*_2_. This is highly possible because the routing strategy positions the proxy nodes for successive packets randomly and far from each other to make the tracing back process more difficult and befuddling. A patient adversary has a higher chance of making progress towards the source node since it stays static at an immediate sender node. It is possible that a packet will arrive at that immediate sender node after sometime of waiting. Also, the all-direction random routing scheme has a longer safety period against a cautious adversary as compared to when it is used against a patient adversary due to its random routing paths. The phantom single-path routing and randomly selected intermediate node routing have a somewhat better performance against a cautious adversary. Shortest path routing scheme has a slightly shorter safety period against a cautious adversary as compared to when it is used against a patient adversary. This is because a cautious adversary is more powerful when used to attack against the short routing paths which are very easy to predict and trace back.

Figure 7 shows attack success rate at a trace time of 900 source packets for different number of nodes in the network. Attack success rate measures the rate of source node traceability for a routing scheme against the back tracing adversary [5]. Attack success rate has an inversely proportional relationship with the safety period of a routing scheme. The higher the safety period and privacy level of a routing scheme, the lower the adversary attack success rate. The figure shows the proposed scheme has a low attack success rate as compared to the other schemes. The figure also shows attack success rate of the proposed scheme decreases at a faster rate as the node intensity increases. This is due the availability of more nodes to create more random routing paths. An increase in number of proxy nodes and neighboring nodes allow different routes to be created for each packet when randomization factor *R_F_* is applied. While using randomization factor *R_F_*, having more number of nodes in proxy regions *P*_2_, *P*_3_ and *P*_4_ will make the routing paths more random and improve the path diversity between successive packets. For example, assume the source node *S*_2_ from Figure 4 has *n* neighboring nodes, the probability of selecting a particular neighboring node is 1/n. If region *P*_2_, has *m* proxy nodes, the probability of *S*_2_ selecting a particular proxy node in region *P*_2_ is 1/m. If region *P*_3_, has *k* proxy nodes, the probability of *S*_2_ selecting a particular proxy node in region *P*_3_ is 1/k. Then, the total number of possible routing paths through region *P*_2_ is nm and through region *P*_3_ is nk. Overall, the total number of routing paths for *S*_2_ will be nm+nk=n(m+k). This shows that the total number of routing paths is directly proportional to the number of neighboring nodes and nodes in proxy regions *P*_2_, *P*_3_ or *P*_4_.

Figure 8 shows the cost performance of the routing schemes for delivering the same amount of packets to the sink node. Figure 8a–c show the delivery latency, packet delivery ratio and total energy consumption of the schemes, respectively. The proposed scheme provides stronger privacy at a cost of an acceptable increase in the packet delivery latency and total energy consumption, with a reduced packet delivery ratio, as compared to the shortest path routing, phantom single-path routing and randomly selected intermediate node routing. This is because the proposed scheme uses elongated routing paths which are highly random. However, the proposed scheme is more cost-effective than the all-direction random routing scheme. The all-direction random routing scheme has the highest packet delivery latency and total energy consumption with the lowest packet delivery ratio because it employs multiple sink nodes to route the packets. The packets are randomly routed through any of the four sink nodes with equal probability. After a sink node receives a packet, all sink nodes have to communicate directly to synchronize the packet information. The synchronization process introduces additional latency and energy consumption. Furthermore, the process reduces the packet delivery ratio due to the possible higher number of packet collisions. Figure 8c shows the total energy consumption is higher near the sink region because this region has a bigger load of packets to forward to the sink node. The figure also shows the proposed scheme is more cost-effective than the strategic location-based random routing scheme.

The proposed scheme works well for one directional monitoring applications similar to the one assumed in this paper where the sink node is located towards the network edge. A network operator will have to consider a different configuration for the proxy regions if the position of the sink node is changed within the WSN domain. 

## 7. Conclusions and Future Work

The use of WSNs in monitoring applications requires designing of effective source location privacy routing schemes. This is particularly true when the WSNs are used in safety-critical monitoring applications. This paper presents a two-phase quadrant-based routing scheme to address the source location privacy problem. The scheme provides a dynamic route creation process with tactically positioned random proxy nodes. At each forwarding instance, the scheme employs a randomizing parameter to guarantee vastly random routing paths. Performance of the proposed scheme and other existing schemes were evaluated against the patient and cautious adversary models. Results reveal that the proposed scheme has longer and highly random routing paths which are capable of obfuscating the adversaries to provide stronger source location privacy. Furthermore, the results show that despite the cautious adversary having more computational power as compared to the patient adversary, the proposed scheme can inhibit the cautious adversary from making any momentous progress towards the source node. Overall, the proposed scheme can provide strong source location privacy in all areas of the WSN domain which makes it a great contender for safety-critical monitoring applications. The scheme incurs slightly higher packet delivery costs. Nevertheless, it is more cost-effective than some of the recently proposed routing schemes. As part of future work, other network configurations such as multiple mobile source nodes will be considered.

## Figures and Tables

**Table 1:** Summary of the routing strategies and key features of the schemes.

Scheme	Key Features	Limitations	Strategy for Improvement in Proposed Scheme
Shortest path routing [6]	Packet forwarding algorithm employs a single shortest path between source node and sink.	Packet routes are short and easy for adversary to back trace. Short packet routes are closely related leading to poor privacy.	Use of tactically positioned proxy nodes to elongate packet routes. Use of randomization factor guarantees routes are not closely related.
Phantom single-path routing [8]	Routing through a random phantom node, then, uses a single fixed path between the phantom node and sink node. Always obeys a constant rule to create routing paths.	The single fixed path is implemented using the shortest path routing strategy which provides poor privacy. Applying a constant rule throughout the network causes poor privacy if a source node is near the sink.	Tactically positioned proxy nodes elongate the packet routes. Proxy node selection process is dynamic depending on source node location.
Randomly selected intermediate node routing [10]	Routing through an intermediate node outside a pre-defined region around the source node. Intermediate node forwards packet to sink node through a fixed route. Always obeys a constant rule to create routing paths.	Fixed routes between intermediate nodes and sink node reduce the privacy level. Employing a constant rule throughout the network may create short routes with poor privacy.	Route creation is dynamic through the use of randomization factor. Proxy node selection process is dynamic.
All-direction random routing [11]	Employs multiple sink nodes and packet routing is through random agent nodes.	Use of multiple sink nodes requires the sink nodes to further communicate directly. This introduces additional packet delivery costs. Less random agent node selection process with high probability of using same agent node for successive packets.	Employs a single sink node which is strategically positioned. Use of randomization factor guarantees high probability of using different proxy nodes for successive packets.

**Table 2:** Proxy node selection.

Source Node Location	Proxy Node Selection	
	*R_F_* < *T*	Otherwise
*Quadrant 1*	*P*_2_	*P*_4_
*Quadrant 2*	*P*_4_	*P*_3_
*Quadrant 3*	*P*_4_	*P*_2_
*Quadrant 4*	*P*_3_	*P*_2_

**Table 3:** Network simulation parameters.

Parameter	Value
Network size (m^2^)	2000 × 2000
Number of nodes	2500
*d_min_* (m)	500
Sensor node sensing range (m)	30
Adversary detection range (m)	30
Initial energy (J)	0.5
Threshold distance (*d*_0_) (m)	87
E_elec_(nJ/bit)	50
E_amp_ (pJ/bit/m^4^)	0.0013
E_fs_ (pJ/bit/m^2^)	10
Packet size (bit)	1024
Target monitoring scheme	k-nearest neighbors tracking
